# The Effect of Berberine on Metabolic Profiles in Type 2 Diabetic Patients: A Systematic Review and Meta-Analysis of Randomized Controlled Trials

**DOI:** 10.1155/2021/2074610

**Published:** 2021-12-15

**Authors:** Jing Guo, Hongdong Chen, Xueqin Zhang, Wenjiao Lou, Pingna Zhang, Yuheng Qiu, Chao Zhang, Yaoxian Wang, Wei Jing Liu

**Affiliations:** ^1^Renal Research Institution of Beijing University of Chinese Medicine, and Key Laboratory of Chinese Internal Medicine of Ministry of Education and Beijing, Dongzhimen Hospital Affiliated to Beijing University of Chinese Medicine, Beijing, China; ^2^Department of Endocrinology, Beijng Hepingli Hospital, NO.18th Hepingli North Street, Beijing 100013, China; ^3^Institute of Nephrology, and Zhanjiang Key Laboratory of Prevention and Management of Chronic Kidney Disease, Guangdong Medical University, No. 57th South Renmin Road, Zhanjiang, Guangdong 524001, China

## Abstract

**Objective:**

*Rhizoma Coptidis* is an herb that has been frequently used in many traditional formulas for the treatment of diabetic mellitus (DM) over thousands of years. Berberine, the main active component of *Rhizoma Coptidis*, has been demonstrated to have the potential effect of hypoglycemia. To determine the potential advantages of berberine for diabetic care, we conducted this systematic review and meta-analysis to examine the efficacy and safety of berberine in the treatment of patients with type 2 DM.

**Methods:**

Eight databases including PubMed, Embase, Web of Science, the Cochrane library, China National Knowledge Infrastructure (CNKI), Chinese Biomedical Database (SinoMed), Wanfang Database, and Chinese VIP Information was searched for randomized controlled trials (RCTs) reporting clinical data regarding the use of berberine for the treatment of DM. Publication qualities were also considered to augment the credibility of the evidence. Glycemic metabolisms were the main factors studied, including glycosylated hemoglobin (HbA1c), fasting plasm glucose (FPG), and 2-hour postprandial blood glucose (2hPG). Insulin resistance was estimated by fasting blood insulin (FINS), homeostasis model assessment-insulin resistance (HOMA-IR), and body mass index (BMI). Lipid profiles were also assessed, including triglyceride (TG), total cholesterol (TC), low-density lipoprotein (LDL), and high-density lipoprotein (HDL), along with inflammation factors such as C-reactive protein (CRP), interleukin-6 (IL-6), and tumor necrosis factor-*α* (TNF-*α*). Serum creatinine (Scr), blood urea nitrogen (BUN), and adverse events were applied to evaluate the safety of berberine.

**Results:**

Forty-six trials were assessed. Analysis of berberine applied alone or with standard diabetic therapies versus the control group revealed significant reductions in HbA1c (MD = −0.73; 95% CI (−0.97, −0.51)), FPG (MD = −0.86, 95% CI (−1.10, −0.62)), and 2hPG (MD = −1.26, 95% CI (−1.64, −0.89)). Improved insulin resistance was assessed by lowering FINS (MD = −2.05, 95% CI (−2.62, −1.48)), HOMA-IR (MD = −0.71, 95% CI (−1.03, −0.39)), and BMI (MD = −1.07, 95% CI (−1.76, −0.37)). Lipid metabolisms were also ameliorated via the reduction of TG (MD = −0.5, 95% CI (−0.61, −0.39)), TC (MD = 0.64, 95% CI (−0.78, −0.49)), and LDL (MD = 0.86, 95% CI (−1.06, −0.65)) and the upregulation of HDL (MD = 0.17, 95% CI (0.09, 0.25)). Additionally, berberine improved the inflammation factor.

**Conclusion:**

There is strong evidence supporting the clinical efficacy and safety of berberine in the treatment of DM, especially as an adjunctive therapy. In the future, this may be used to guide targeted clinical use of berberine and the development of medications seeking to treat patients with T2DM and dyslipidemia.

## 1. Introduction

Diabetes mellitus (DM) is a chronic, noncommunicable disease that has become a worldwide threat to public health. The global prevalence of DM has been remarkably increased to 463 million among adults and has been predicted to surge to about 700 million by the year 2045. In 2019, approximately 4.2 million adults had an estimated cause of death related to diabetes and its complications. This comes out to about one death every eight seconds [1]. Though 10% of the global health expenditure is currently spent on DM, this disease remains in the top 4 causes of noncommunicable disease deaths [2]. Thus, it is essential to explore more effective and safe strategies for the prevention and treatment of DM. China currently has the largest number of type 2 diabetic patients, so this condition has become a leading public health challenge in China [3].

In addition, with the development of society and the change of diet structure, the characteristics of the diabetic population are changing. The classic symptoms, like polydipsia, polyphagia, polyuria, and weight loss, are less often recognized in those with type 2 diabetes mellitus (T2DM). It has been reported that 44% of type 2 diabetic respondents had no classic symptoms in the previous year [4]. Moreover, patients with T2DM are more likely to be overweight or obese, which indicates insulin resistance and dyslipidemia along with hyperglycemia [5, 6]. For T2DM patients, glycemic or lipid values and their variability can significantly predict all-cause mortality and the occurrence of complications, including micro- and macrovascular complications [7, 8]. Growing evidence has indicated that chronic inflammation, hyperglycemia, and dyslipidemia are all involved in insulin resistance, pathogenesis of T2DM, and systematic diabetic complications [9, 10]. In T2DM patients, IL-6 and TNF-*α* levels are strikingly increased. This is also associated with a downregulation of several drug metabolizing enzymes, which leads to poor drug effects [11].

Huanglian (*Rhizoma Coptidis*) is an herb that has been frequently used in many traditional formulas for the treatment of T2DM. This treatment has been used for thousands of years. Alkaloids from Huanglian have been widely used for the treatment of diabetes and hyperglycemia with inconspicuous toxicities and side effects [12]. Among the different alkaloids, berberine is an important lead compound with a wide spectrum of pharmacological activities, including antitumor, anti-inflammatory, hypoglycemic, hypolipidemic, and antiobesity effects [13]. Additionally, evidence has shown that berberine regulates the gut microbiota as well [14, 15], which is closely associated with systematic inflammation that adds to the progression of T2DM. Systematic reviews have previously reported the efficacy of berberine on the regulation of glycemic and lipid metabolisms [16, 18]. However, its comprehensive effects on glycemic metabolisms, lipid profiles, and inflammation factors of patients with T2DM have not been well evaluated. Since berberine is the main active component of *Rhizoma Coptidis*, we conducted this meta-analysis to comprehensively evaluate the efficacy and safety of berberine and *Rhizoma Coptidis* on T2DM.

## 2. Methods

### 2.1. Search Strategy and Study Selection

This study was performed according to the Cochrane Handbook for Systematic Reviews of Interventions and was reported according to Preferred Reporting Items for Systematic reviews and Meta-Analyses (PRISMA) [19]. The protocol has been registered on PROSPERO as CRD42020155086.

The literature research was conducted with the use of the following eight databases with no time restriction: PubMed (up to March 29_,_ 2021), Embase (up to March 29, 2021), Web of Science (ended up to March 29, 2021), the Cochrane library (up to March 29, 2021), China National Knowledge Infrastructure (CNKI) (up to April 14, 2021), Chinese Biomedical Database (SinoMed) (up to April 14, 2021), Wanfang Database (up to April 14, 2021), and Chinese VIP Information (up to April 14_,_ 2021). The MeSH and free-text terms were applied based on the characteristics of specific database as follows: berberine, huanglian, *Rhizoma Coptidis*, traditional Chinese medicine, diabetes mellitus, insulin resistance, and randomized controlled trials. No restrictions on publication language or date were set. The terms were searched as “diabetes mellitus” OR “insulin resistance” OR “metabolic syndrome” OR “Xiaoke syndrome” AND “TCM” OR” Traditional Chinese Medicine” OR “herbal medicines” OR “plant medicines” OR “berberine” OR “huanglian” OR “ Coptidis “ AND “randomized controlled trial” OR “controlled clinical trial” OR “random” OR “double-blind” OR “single-blind.” The full electronic search strategy for PubMed was provided in Supplementary Files 1 according to the search history.

The included clinical studies fulfilled the following criteria:

1. Types of studies: only RCTs were eligible for this review. Single-blinded and open label trials were also considered. The sample size was greater than or equal to 60 participants, and the intervention duration was no less than four weeks
2. Types of participants: adults aged 18 years or older with T2DM or prediabetes were included
3. Types of interventions: intervention with berberine or *Rhizoma Coptidis* was considered. The control intervention included placebo, life interventions such as changes in exercise or dietary habits, or any active antihyperglycemia intervention
4. Types of outcomes: primary outcomes included glycosylated hemoglobin (HbA1c), fasting plasma glucose (FPG), and 2-hour postprandial plasma glucose (2hPG)

Secondary outcomes included insulin resistance and the associated index of fasting plasma insulin, homeostasis model assessment-insulin resistance (HOMA-IR), and body mass index (BMI). Lipid profiles include triglyceride, total cholesterol, high-density lipoprotein (HDL), and low-density lipoprotein (LDL) along with inflammation markers, such as C-reactive protein.

Safety outcomes included the incidence of adverse reactions and adverse reactions.

The excluded criteria were as follows:

1. Other TCM treatments applied in either the treatment or control group
2. If the publication was a review, an abstract, a protocol, or had no outcomes of interest, it would not be considered
3. Full texts were not available

### 2.2. Data Collection and Analysis

Titles and abstracts were screened independently by two investigators. The NoteExpress 3.4 literature management software was used to screen references. It was suggested to reviewers to contact the author for the complete information if this was indicated. If there was a disagreement, another reviewer would help determine a solution. Kappa statistics were employed to analyze the consistency of the results.

Data extraction was carried out independently by two authors using standard data extraction forms that included participant details, the interventions used in both treatment and control group, and the primary and secondary outcomes. Where more than one publication of one study existed, reports were grouped together and the publication with the most complete data was used in the analyses.

### 2.3. Assessment of Risk of Bias in Included Studies

Two reviewers independently assessed the quality of each article, using the risk of bias assessment tool in the Cochrane. Another reviewer was asked to help decide if there was a disagreement.

### 2.4. Measures of Treatment Effect

Categorical outcomes were expressed as risk ratio (RR) with 95% confident intervals (CI), while continuous measurement was pooled by the standardized mean difference (SMD) or mean difference (MD). If data were missing or unclear, it was suggested that reviewers contact the authors of studies to request this information.

### 2.5. Assessment of Heterogeneity

Heterogeneity was analyzed using a Chi^2^ test with an alpha of 0.1. The *I*^2^ statistic was used for statistical significance. When *I*^2^ was less than or equal to 50% and *P* > 0.1, the heterogeneity was acceptable. In addition, when *I*^2^ was greater than 50% and *P* < 0.1, the heterogeneity among the trials was significant.

### 2.6. Data Synthesis and Analysis

Revman 5.4 software was used to conduct the data synthesis and analysis. In the analysis, pooling data was processed as overall RR and/or MD. A random effects model was applied if there was any heterogeneity observed. If pooling was not possible, the data were summarized descriptively.

Subgroup analysis was planned to explore the source of heterogeneity according to the differences of treatment methods among trials. Sensitivity analyses were conducted using a leave-one-out method to identify studies contributing to significant heterogeneity, which had an *I*^2^ value of greater than 50%. Funnel plots were planned to assess publication bias as well.

## 3. Results

**Figure 1:**
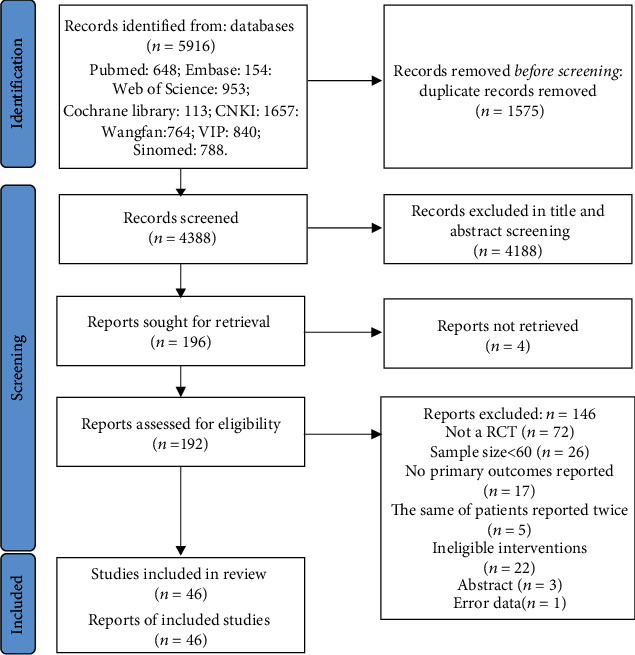
Flow diagram.

A total of 46 clinical trials were considered and included in the quantitative meta-analysis (Figure 1). As to the literature screening and selection, the result of kappa statistics was *k* = 0.785, indicating a good consistency. Of these, 38 were related to berberine in the treatment of T2DM, and four [20–23] were for prediabetes. Two trials [24, 25] were aimed at evaluating the effectiveness and safety of *Rhizoma Coptidis* in the treatment of T2DM, and two [26, 27] were for root dry extracts including berberine. The publication time of the included studies was from 2004 to 2021.

A total of 4,158 participants were enrolled, including 2,063 participants in the experimental group and 2,095 participants in the control group. The intervention duration ranged from 4 weeks to 6 months. Eleven trials compared berberine with placebo or none, while four trials compared berberine with metformin. Specific characteristics of the included trials are illustrated in Table 1.

**Figure 2:**
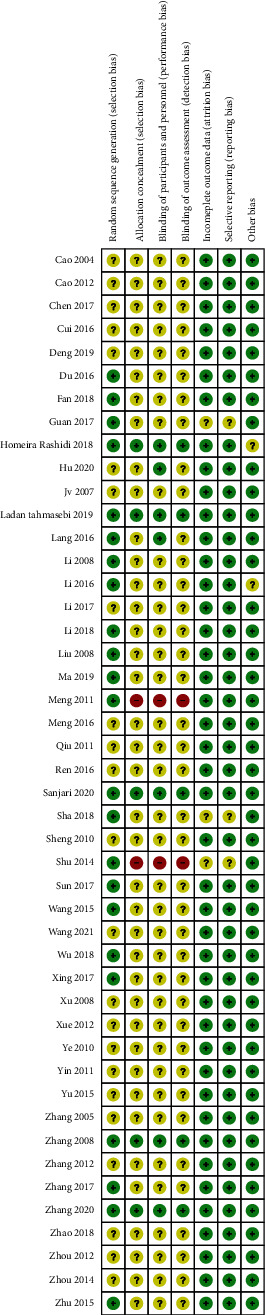
Risk of bias of assessment. The quality of each article independently using the risk of bias assessment tool in the Cochrane.

All the included studies were RCT designs and twenty-two of them provided information on random sequence generation. Five studies reported information on allocation concealment procedures and the blindness of outcome assessments. Five studies were conducted with blinding of both participants and personnel. No risks of incomplete outcome data or selective reporting were found for any of the recruited studies. The risks of bias assessment across the recruited studies are illustrated in Figure 2. To comprehensively illustrate the effect of berberine for diabetes, glucose metabolism-related index (HbA1c, FPG, and 2hPG), insulin resistance-related index (FINS, HOMA-IR, and BMI), lipid profiles (TC, TG, HDL, and LDL), and inflammation index (CRP, IL-6, and TNF-*α*) were reported, along with the safety including effect of berberine on serum creatinine (SCR), blood urea nitrogen (BUN), and any associated adverse events.

### 3.1. Berberine for Glucose Metabolism of T2DM

Forty trials, 45 trials, and 36 trials provided the data of HbA1c, FPG, and 2hPG, respectively. The meta-analyses showed that berberine remarkably decreased the HbA1c level (MD = −0.75, 95% (−1.00, −0.51); *P* < 0.05; *I*^2^ = 98%, 95% CI (0.97, 0.98)), the FPG level (MD = −0.89, 95% CI (−1.13, −0.64), *P* < 0.05, *I*^2^ = 97, 95% CI (0.96, 0.97)), and the 2hPG level (MD = −1.31, 95% CI (−1.69, −0.93), *P* < 0.05, *I*^2^ = 96%, 95% CI (0.96, 0.97)).

**Figure 3:**
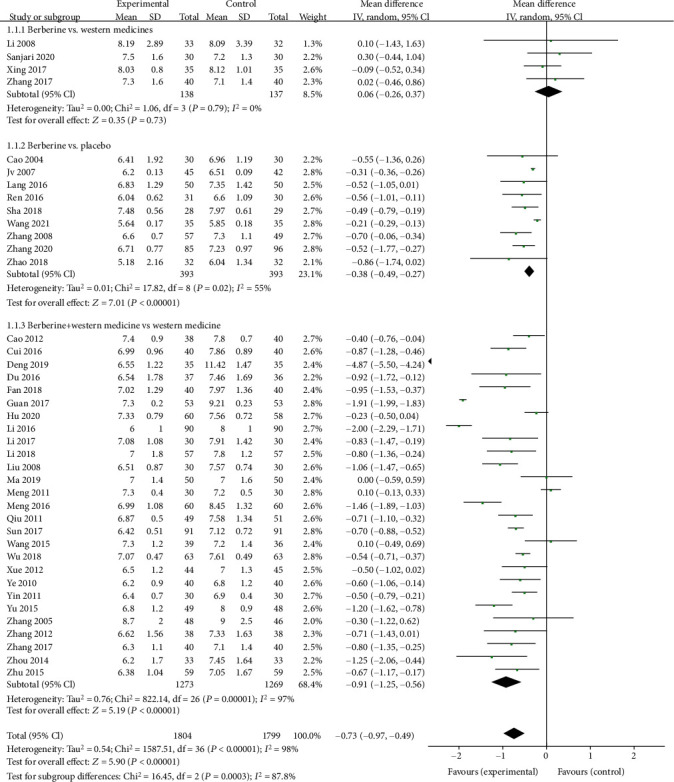
Meta-analysis of the effect of berberine on glycosylated hemoglobin (HbA1c).

**Figure 4:**
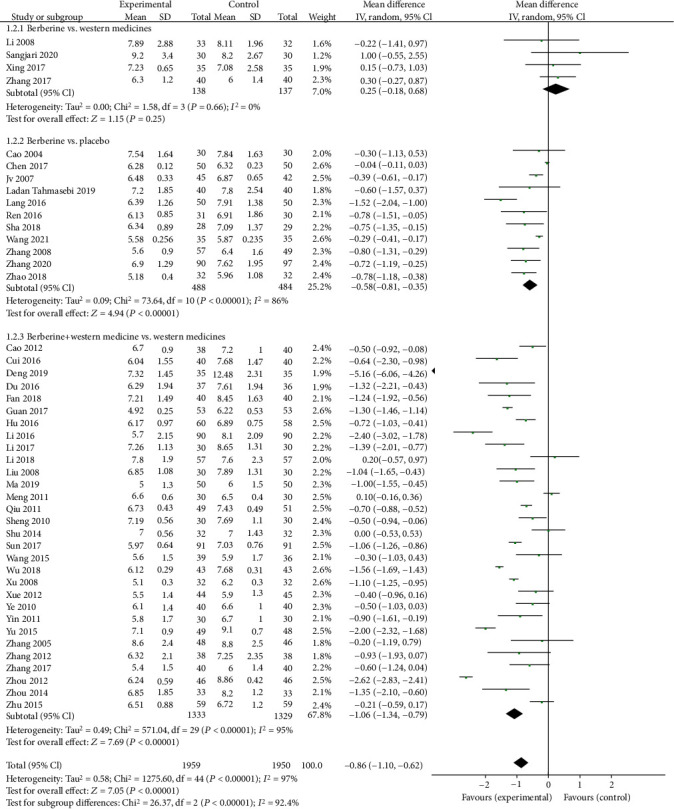
Meta-analysis of the effect of berberine on fasting plasma glucose (FPG).

**Figure 5:**
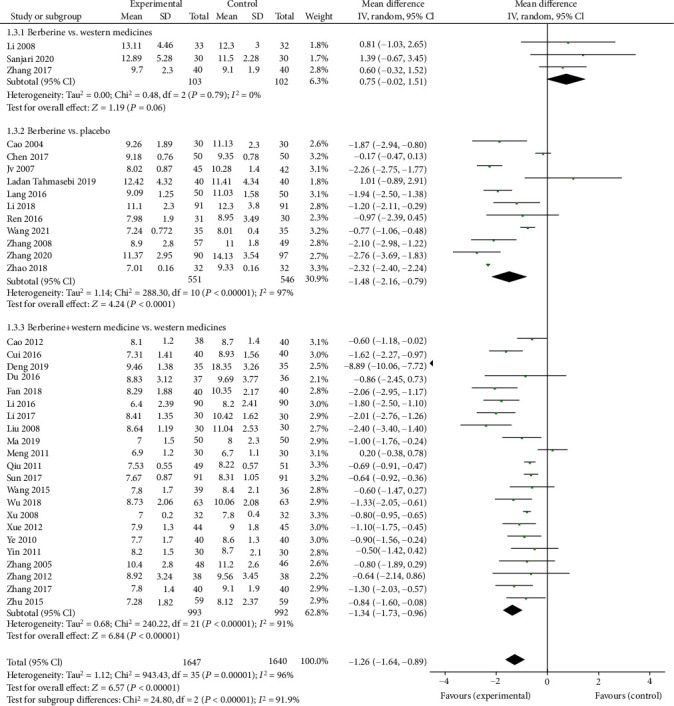
Meta-analysis of the effect of berberine on 2-hour postprandial plasma glucose (2hPG).

Subgroup analysis showed that berberine could slightly lower the HbA1c level (MD = −0.38, 95% CI (−0.49, −0.27), *P* < 0.05) and the FPG level (MD = −0.58, 95% CI (−0.81, −0.35), *P* < 0.05) but remarkably reduce the 2hPG level (MD = −1.48, 95% CI (−2.16, −0.79), *P* < 0.05) when it was used alone. When combined with antidiabetic agents, berberine strikingly reduce the HbA1c level (MD = −0.91, 95% CI (−1.25, −0.56), *P* < 0.05), the FPG level (MD = −1.06, 95% CI (−1.34, −0.79), *P* < 0.05), and the 2hPG level (MD = −1.34, 95% CI (−1.73, −0.96), *P* < 0.05). However, compared with Western medicine, there was no significant difference observed in the HbA1c level, the FPG level, or the 2hPG level when berberine was applied alone (Figures 3–5).

### 3.2. Berberine for Insulin Resistance-Associated Index of T2DM

**Figure 6:**
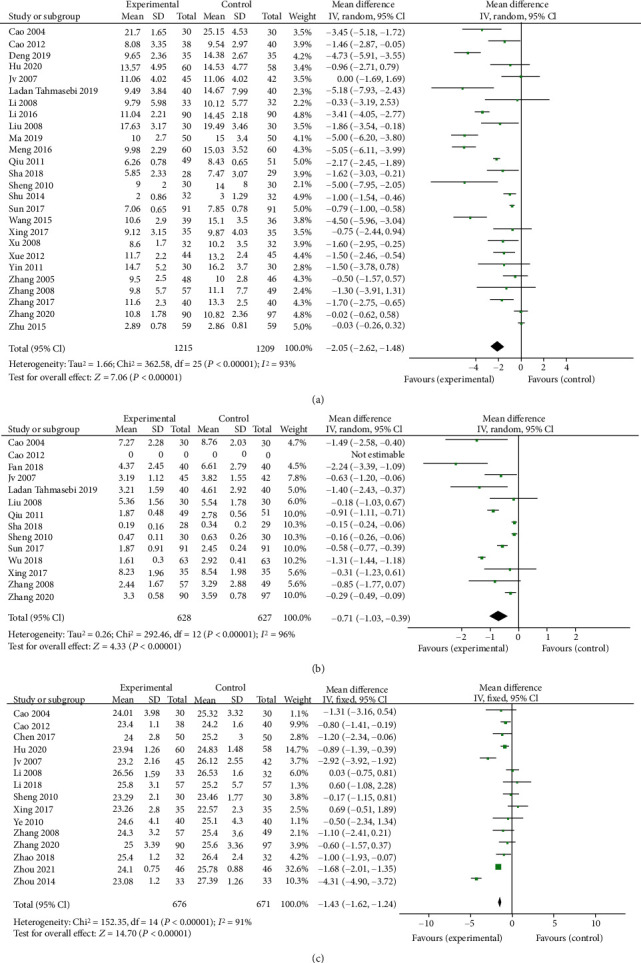
Meta-analysis of the effect of berberine on insulin resistance-associated index. (a) Fasting plasma insulin (FINS). (b) Homeostasis model assessment-insulin resistance (HOMA-IR). (c) Body mass index (BMI).

Figure 6 shows the efficacy of berberine on the FINS level, the HOMA-IR level, and the BMI level of T2DM patients. The FINS concentration of the trial group decreased by 2.05 (95% CI (−2.62, −1.48), *P* < 0.05, *I*^2^ = 93%, 95% CI (0.91, 0.95)), the HOMA-IR level of the berberine group decreased by 0.71 (95% CI (−1.03, −0.39), *P* < 0.05, *I*^2^ = 96%, 95% CI (0.94, 0.97)), and the BMI level of the berberine group decreased by 1.07 (95% CI (−1.76, −0.37), *P* < 0.05, *I*^2^ = 91%, 95% CI (0.87, 0.94)).

### 3.3. Berberine for Lipid Profiles of T2DM

**Figure 7:**
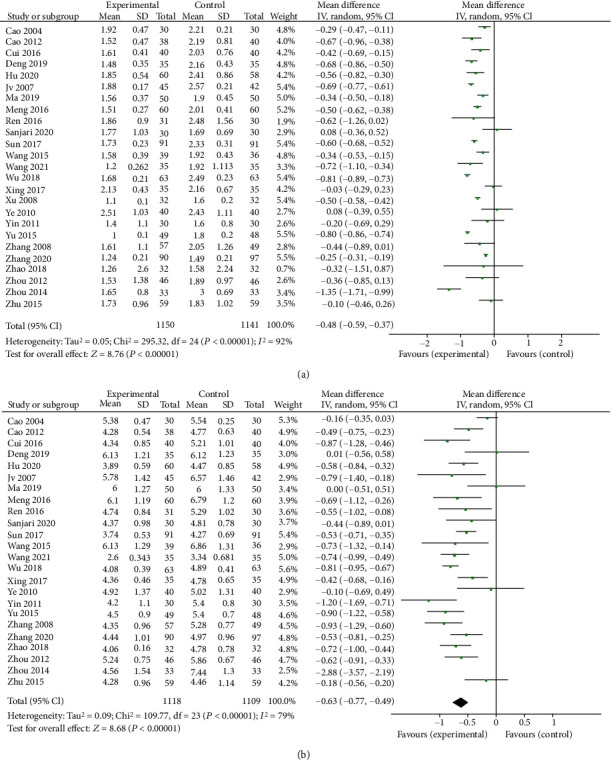
Meta-analysis of the effect of berberine on triglyceride (a) and total cholesterol (b).

**Figure 8:**
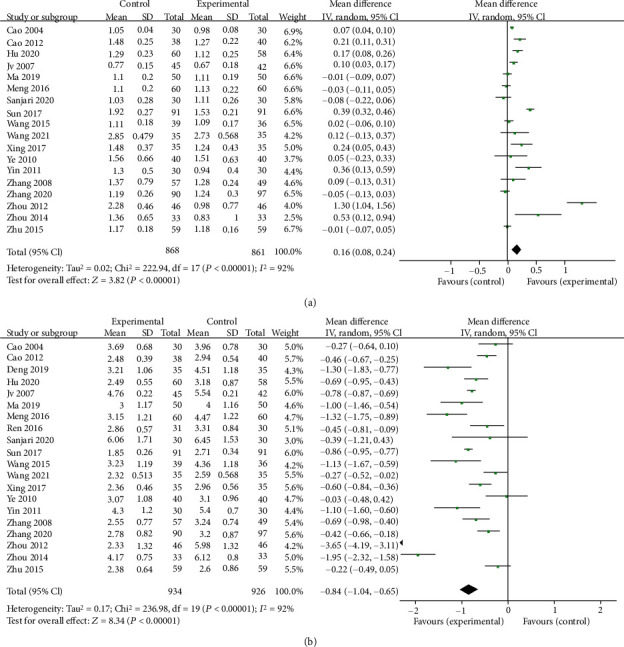
Meta-analysis of the effect of berberine on high-density lipoprotein (a) and low-density lipoprotein (b).

The pooled results of berberine efficacy in the treatment of T2DM on lipid profiles are shown in Figures 7 and 8. Twenty-four trials were assessed on TG (Figure 7(a)), and the result showed that berberine significantly lowered the TG level in patients with T2DM (MD = −0.5, 95% CI (−0.61, −0.39), *P* < 0.05, *I*^2^ = 92%, 95% CI (0.89, 0.94)). Pooled results of 25 trials showed that the TC concentration of the berberine group decreased by 0.64 (95% CI (−0.78, −0.49), *P* < 0.05, *I*^2^ = 79%, 95% CI (0.69, 0.86)) (Figure 7(b)). There was a slightly upregulated tendency in HDL (MD = 0.17, 95% CI (0.09, 0.25), *P* < 0.05, *I*^2^ = 92%, 95% CI (0.89, 0.95)) (Figure 8(a)), as compared to the control group and the LDL concentration of the berberine group decreased by 0.86 (95% CI (−1.06, −0.66), *P* < 0.05, *I*^2^ = 92%, 95% CI (0.89, 0.94)) (Figure 8(b)).

### 3.4. Berberine for Inflammation Factors of T2DM

Pooled results of six clinical trials proved that berberine markedly lowered the CRP levels in patients with T2DM (SMD = −2.13, 95% CI (−2.98, −1.28), *P* < 0.05, *I*^2^ = 96%, 95% CI (0.94, 0.97)).

Six trials were considered for the analysis of the efficacy of berberine on IL-6 levels. The IL-6 concentration in the trial group decreased by 1.83 (95% CI (−3.05, −0.61), *P* = 0.003; *I*^2^ = 97%, 95% CI (0.95, 0.98)).

**Figure 9:**
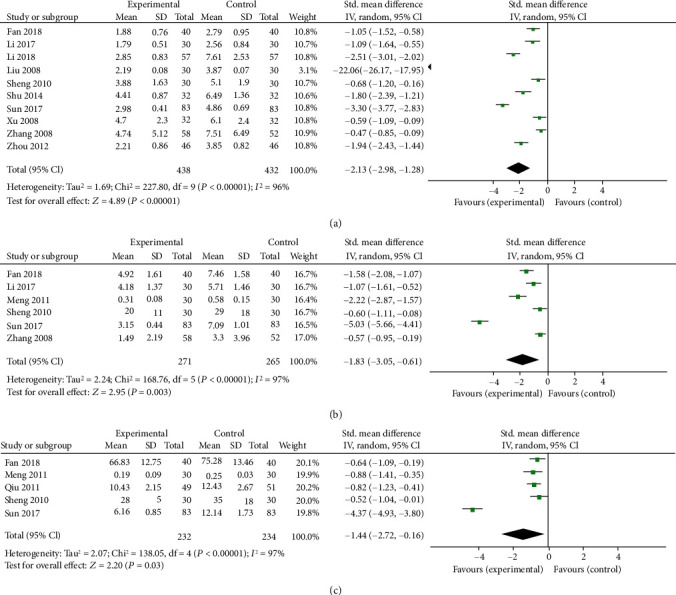
Meta-analysis of the effect of berberine on inflammation factors. (a) C-reaction protein (CRP). (b) Interleukin-6 (IL-6). (c) Tumor necrosis factor-*α* (TNF-*α*).

Among them, five reported the efficacy of berberine on TNF-*α*, and the results found that berberine reduced the level of TNF-*α* in patients with T2DM to some extent (SMD = −1.44, 95% CI (−2.72, −0.16), *P* = 0.03, *I*^2^ = 97%, 95% CI (0.95, 0.98)) (Figure 9).

### 3.5. Safety of Berberine on Patients with T2DM

**Figure 10:**
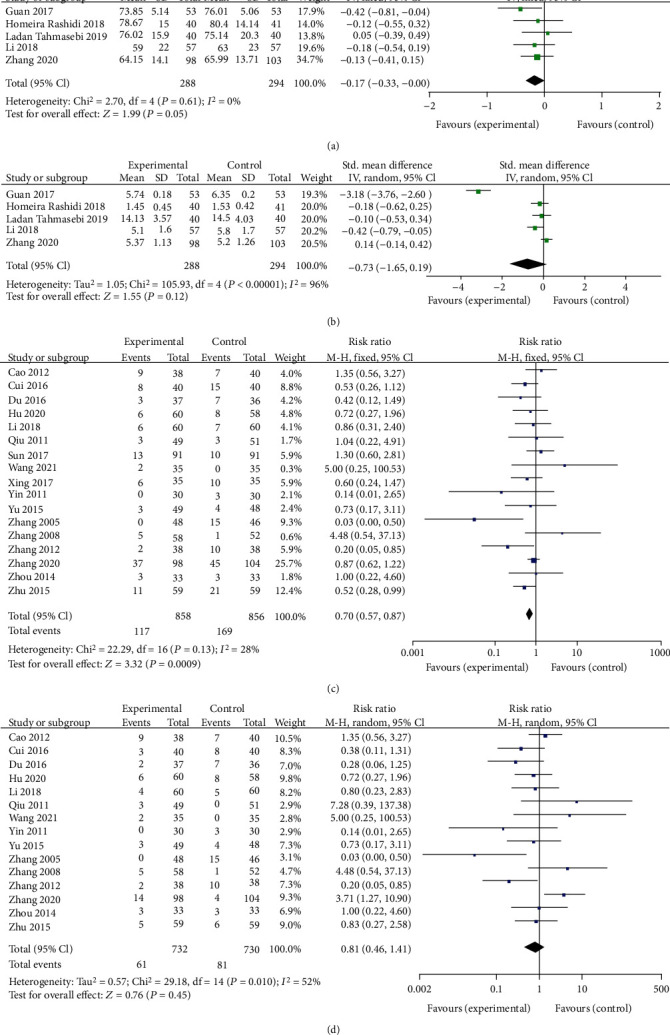
Meta-analysis of the safety of berberine in the treatment of diabetes mellitus. (a) Serum creatinine (Scr). (b) Blood urea nitrogen (BUN). (c) Total adverse events. (d) The gastrointestinal adverse events.

Figure 10(a) shows the effects of berberine on Scr in patients with T2DM. There were a total of 288 volunteers in the trial group and 294 in the control group. The Scr concentration of the berberine group decreased by 2.02 (95% CI (−3.63, −0.42), *P* = 0.01, *I*^2^ = 0%, 95% CI (0, 0.86)). Berberine was found to have a beneficial effect on Scr.

Figure 10(b) illustrates the effects of berberine on BUN. There were a total of 288 volunteers in the trial group and 294 in the control group. Compared to the control group, berberine had no significant effect on the BUN level (SMD = −0.29, 95% CI (−0.69, −0.11), *P* = 0.16, *I*^2^ = 97%, 95% CI (0.94, 0.98)).

The incidence of adverse events (AEs) was used to assess the safety of berberine in 17 studies, including a total of 858 patients in the berberine group and 856 in the control group. Pooled results of 17 trials showed that berberine applied for the treatment of T2DM appeared to have better safety compared to the control group in the incidence of AEs (RR = 0.70, 95% CI (0.57, 0.87), *P* = 0.0009, *I*^2^ = 28%, 95% CI (0, 0.6)) (Figure 10(c)). The main reported adverse events of berberine treatment were gastrointestinal responses like diarrhea, abdominal distention, or constipation. Among the 17 trials, 15 specifically reported the number of gastrointestinal AEs, which included 732 volunteers in the trial group and 730 in the control group. These results demonstrated that berberine did not have more gastrointestinal AEs as compared to the control group (RR = 0.81, 95% CI (0.46, 1.14), *P* = 0.45, *I*^2^ = 52%, 95% CI (0.13, 0.73)) (Figure 10(d)). The pooled results demonstrated that berberine was generally safe as a complementary or alternative therapy for the treatment of T2DM.

### 3.6. Publication Bias

**Figure 11:**
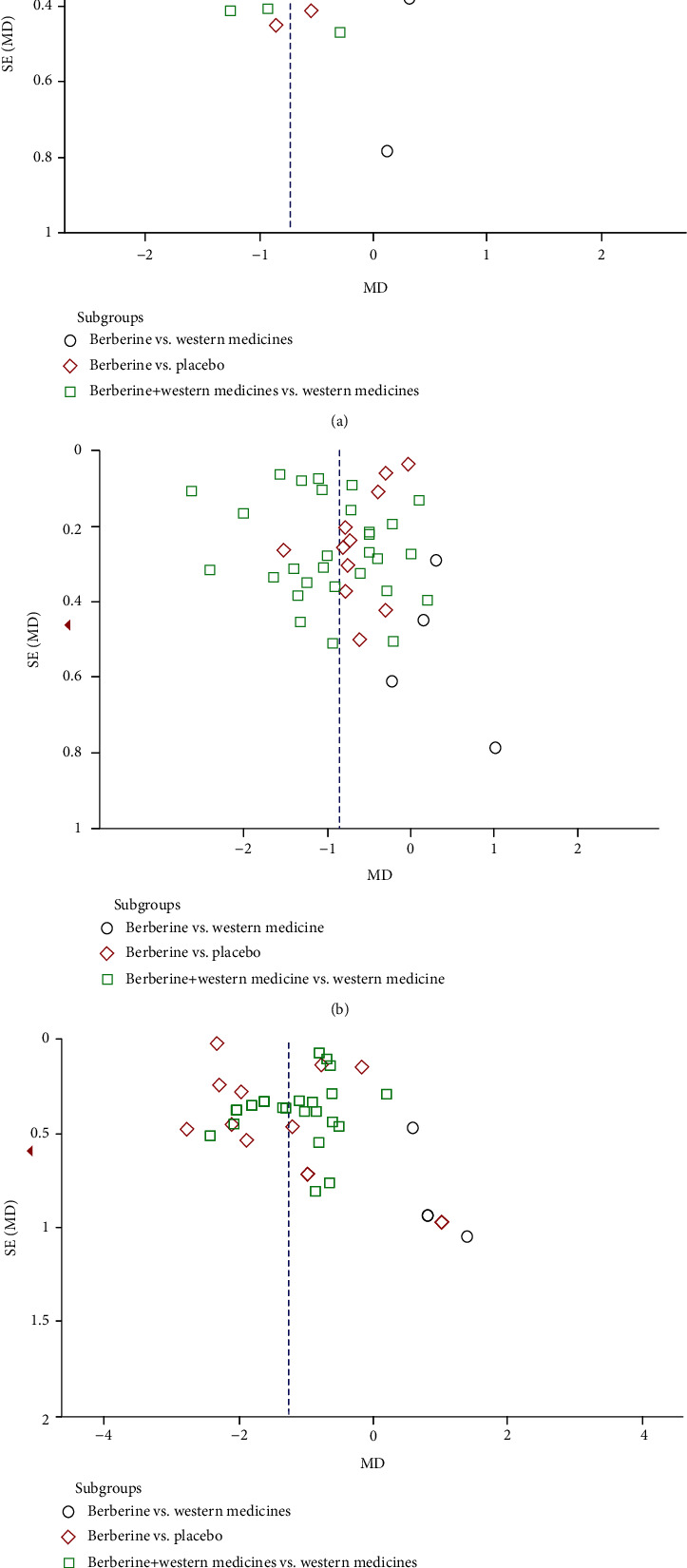
Funnel plots of comparison. A funnel plot was used to evaluate potential publication bias. Comparison in HbA1c, FPG, and 2hPG was conducted by funnel plots.

A funnel plot was used to evaluate potential publication bias. Comparisons of HbA1c, FPG, and 2hPG were conducted using funnel plots. Approximately symmetrical dispersion points suggested rare publication bias. These results are shown in Figure 11.

### 3.7. Sensitivity Analysis

According to the different interventions, daily dosage of berberine applied, the intervention duration, and disease courses, we conducted the subgroup analyses for main outcomes including HbA1c, FPG, and 2hPG (Supplementary Files 2). Results found that different interventions were the source of heterogeneity. Mutual conversion between a random-effects model and a fixed-effect model was conducted as a sensitivity analysis for testing the stability of the research. The results showed that the *I*^2^ value did not change in the mutual conversion. The results of meta-analyses were not changed either. This suggests that our findings were stable.

## 4. Discussion

In the current systematic review, we evaluated the efficacy and safety of berberine for the treatment of T2DM. Our findings suggested that berberine, used along or combined with antidiabetic agents, significantly improved glucose and lipid metabolisms along with inflammation markers.

Berberine showed effectiveness in lowering blood glucose comparable with metformin. As an adjunctive therapy, berberine presented better reduction of HbA1c, FPG, and 2hPG. In recent years, three meta-analyses [17, 66, 67] were conducted to explore the effects of berberine for the treatment of T2DM. The latest study was published in 2019, which first performed subgroup analyses to examine the source of heterogeneity and identify the potential factors which likely determine the effects of berberine. These results showed that berberine seemed to achieve better effects on glucose levels when patients aged less than 60 years were treated with a daily dosage of 1.5–2 g. After adding new research with limited sample size and intervention durations in the included criteria, our results suggested that low-dose berberine (< 1 g/d) achieve promising effects of FPG, especially for those patients with a disease duration of no more than five years. The efficacy of berberine seemed to decrease with an increased treatment course. Low and medium doses of (1–2 g) berberine showed the same effects on reducing HbA1c and 2hPG. Better efficacy on 2hPG was observed in patients with an intervention duration of no less than 12 weeks. For HbA1c, the highest level of efficacy was observed with an intervention duration of no more than eight weeks and less than twelve weeks, especially for those patients with T2DM that have had this condition for 5–10 years. It appeared that the early application of berberine mainly functioned to reduce FPG. With prolonged intervention duration and disease progression, this effect was mainly observed as the reduction of 2hPG.

The regulation of berberine on blood homeostasis is partly due to the improvement of insulin resistance, the hallmark of T2DM, which is given rise to obesity. Systematic reviews showed that berberine improved obesity parameters including BMI [68, 69]. Meanwhile, a case-control clinical trial reported that the HOMA-IR level of T2DM decreased by 73% with 500 mg (×3 daily) berberine for 3 months [70]. Our results showed that berberine remarkably lower fasting blood insulin, improve HOMA-IR, and decrease BMI, which demonstrated the advantages of berberine on improving insulin resistance.

Evidence suggests that clinically tested lipid-lowering nutraceuticals including berberine could be safely used to improve lipid levels in patients with mild-to-moderate dyslipidemia [71]. Our work also showed the plasma lipid profiles of diabetic patients were improved by berberine intake. The efficacy of berberine on dyslipidemia has been widely researched. Whether used alone or combined with other therapies, meta-analyses [16, 72] suggested that berberine improve obesity and hyperlipidemia by reducing TG, TC, and LDL and increasing HDL in the setting of several metabolic disorders along with improving glucose metabolism. This is consistent with the current meta-analysis specific to T2DM, which showed a remarkable lowering of TC, TG, and LDL, along with moderate upregulation of HDL.

In T2DM, obesity and dyslipidemia bring about low-grade inflammation and factor like IL-6 and TNF-*α* levels were found to be strikingly increased. This was associated with a downregulation of several drug metabolizing enzymes, which led to poor drug effect. Berberine has been demonstrated as a chronic inflammation regulator as well [73]. A meta-analysis proved that berberine supplementation ameliorates the state of chronic inflammation by lowering the serum level of CRP [74]. Our meta-analysis illustrated that berberine significantly downregulates CRP, IL-6, and TNF-*α*. This indicates that berberine as an additional therapy shows synergistic benefits with hypoglycemic agents. Above all, berberine is suggested to be applied to diabetic patients with insulin resistance and dyslipidemia, especially for those patients newly diagnosed with T2DM accompanied by obesity and dyslipidemia.

Regarding safety, our work demonstrated that berberine had no toxic effects on Scr and BUN. In addition, this treatment did not increase the risk of serious adverse events when the routine dosage ranged from 0.6 g to 1.5 g.

Berberine has been demonstrated to have comparable effects in the treatment of T2DM with antidiabetic drugs like metformin that display multiple targets and pathways. For patients with T2DM, the main function of berberine is as a SIRT1 or AMPK agonist to mimic energy restriction [75–77] and to target on NF-*κ*B [78] to improve insulin resistance and inflammation as well as to alleviate the activation of ox-LDL-induced macrophages [79]. In addition, berberine stabilizes LDL receptor mRNA to increase the clearance rate of plasma LDL [80]. This corresponds to its effects of improving insulin resistance and the regulation of glycemic and lipid metabolisms. As we all know, dyslipidemia and inflammation are risk factors of micro- and macrovascular leisures, our meta-analysis suggest that berberine has potential benefits on diabetes with cardiovascular and chronic kidney disease, which has been reported in preclinical studies [81, 82].

Excessive statistical heterogeneity was induced by several factors in our work. First, different antidiabetic agents used as controls also had a distinct influence on the outcomes. Different dosage levels of berberine and the duration of treatment may have resulted in inconsistent efficacy and different disease courses. To account for these, subgroup analyses were used to assess primary outcomes, including HbA1c, FPG, and 2hPG. The results showed that the different interventions in the control groups seemed to be a potential impact factor of heterogeneity. Meanwhile, the literature qualities may have influenced the results. In the future, the association of each factor with the effect of berberine for the treatment of T2DM patients should be quantified by metaregression analysis.

## 5. Strengths and Limitations of the Current Study

Compared to previous meta-analyses, the current study included 46 trials and comprehensively showed the efficacy and safety of berberine for the treatment of T2DM. Subgroup analyses were also conducted to clarify how berberine is used. Additionally, GRADE criteria (Supplementary Files 3) were applied to determine the certainty in the estimate of effect for primary outcomes.

There are some limitations of this review. First, most of the trials were conducted among Chinese patients, which limited the widespread application of this data. Second, excessive statistical heterogeneity appeared in some comparisons; however, the primary source of heterogeneity could not be determined. Third, literature qualities were uneven, although the included trials were RCTs. Many of these studies did not report the methods of blinding and allocation concealment. Lastly, more information on the long-term intervention of berberine for the treatment of T2DM is needed to assess the occurrence risk of diabetic complications.

In conclusion, berberine positively regulated glucose metabolism and lipids, improving insulin resistance and inflammation in patients with T2DM. Thus, berberine was recommended as an adjunctive therapy for T2DM.

## Figures and Tables

**Table 1:** Specific characteristics of included trials.

Study	Participants	No. (T/C)	Age	Sex (M/F)	Course (year)	Intervention	Control	Duration (weeks)	Outcomes
Zhang et al. 2020 [15]	T2DM	98/103	T: 53 (42–61) C: 54 (46–61)	T: 59/39 C: 61/42	Newly diagnosed	Berberine 0.5 g bid	Placebo	12	①②③④⑤⑥⑦⑧⑨⑩
Hu 2020 [28]	T2DM	60/58	T: 43.21 ± 8.14 C: 42.85 ± 6.23	T: 34/26 C: 30/28	T: 2.31 ± 1.06 C: 2.61 ± 0.84	Berberine 0.3 g tid Metformin 0.5 g tid	Placebo Metformin 0.5 g tid	24	①③④⑤⑥⑦⑧⑨⑩
Sanjari et al. 2020 [26]	T2DM	42/38	T: 51.8 ± 9.3 C: 46.5 ± 10	T: 18/24 C: 7/31	T: 4.5 ± 0.25 C: 4.25 ± 0.42	Berberis integerrima root extract 480 mg	Metformin 1 g/d	12	①②③⑥⑦⑧⑨
Li et al. 2021 [20]	Prediabetes	35/35	T: 54.11 ± 8.14 C: 42.85 ± 6.23	T: 22/13 C: 17/18	—	Berberine 0.3 g tid	Life intervention	24	①②③⑥⑦⑧⑨⑩
Tahmasebi et al. 2019 [27]	T2DM	40/40	T: 54.05 ± 8.00 C: 53.07 ± 7.74	T: 17/23 C: 15/25	Less than 10 years	1000 mg dry extract and 157.3 mg berberine	Placebo	6	①②④⑥⑦⑧⑨⑩
Ma 2019 [24]	T2DM	50/50	T: 50.8 ± 3.6 C: 50.2 ± 4.2	T: 31/19 C:28/22	T: 1.77 ± 0.57 C: 1.81 ± 0.53	C+Huanglian 3 g tid	Metformin 0.5 g tid	8	①②③④⑥⑦⑧⑨
Deng et al. 2019 [29]	T2DM	35/35	T: 54.6 ± 2.5 C: 55.3 ± 2.6	T:23/12 C:21/14	T: 6.04 ± 2.43 C: 6.61 ± 2.75	C+berberine 0.3 g tid	Glipizide 5 mg tid	12	①②③④⑥⑦⑧⑨
Chen et al. 2018 [30]	T2DM	40/40	T: 53.27 ± 8.15 C: 52.71 ± 7.89	T: 22/18 C: 24/16	T: 5.59 ± 3.74 C: 5.64 ± 3.58	C+berberine 0.5 g tid	Metformin 0.5 g tid	12	①②③⑩
Wu 2018 [31]	T2DM	63/63	T: 58.61 ± 8.37 C: 58.43 ± 8.26	T: 37/26 C: 38/25	NA	C+berberine 0.2 g tid	Metformin 0.5 g tid	12	①②③⑤⑥⑦⑧⑨⑩
Rashidi et al. 2018 [32]	T2DM	40/41	T: 50.18 ± 4.22 C: 45.12 ± 9.55	T: 14/28 C: 19/22	T: 4.5 ± 1.8 C: 4.3 ± 2.04	Berberine 0.5 g bid	Placebo	4	①②④⑥⑦⑧⑨⑩
Li et al. 2018 [33]	T2DM	57/57	T: 53 ± 15 C: 57 ± 12	T: 22/35 C: 24/33	T: 5.92 (1.92, 8) C:7.25 (2, 10.2)	C+berberine 300 mg tid	Antidiabetic agents	24	①②③⑤⑥⑦⑩
Sha et al. 2018 [34]	T2DM	30/30	T: 58.79 ± 12.27 C: 60.46 ± 11.73	T: 15/13 C: 14/15	T: 7.08 ± 2.68 C: 8.68 ± 4.13	Berberine 500 mg tid	NA	12	①③④⑩
Zhao 2018 [22]	Prediabetes	32/32	T: 54.7 ± 0.8 C: 54.9 ± 0.16	T: 24/8 C: 26/6	—	Berberine 0.3-0.5 g tid	Life intervention	12	①②③⑤⑥⑦
Guan et al. 2017 [35]	T2DM	53/53	T: 65.74 ± 3.85 C: 65.28 ± 3.29	T: 29/24 C: 28/25	T: 6.14 ± 2.44 C: 6.25 ± 2.41	C+berberine 0.5 g tid	Metformin 0.5 g tid	4	①③
Zhang 2017 [36]	T2DM	40/40	T: 58.24 ± 6.15 C: 58.13 ± 6.24	T: 21/19 C: 23/17	T: 5.8 ± 1.9 C: 5.7 ± 1.8	Berberine 3 g tid	Metformin 750 mg/d	12	①②③④
		40/40	T: 58.91 ± 6.58 C: 58.13 ± 6.24	T:22/18 C:23/17	T: 6.0 ± 1.7 C: 5.7 ± 1.8	C+berberine 3 g tid	Metformin 750 mg/d	12	①②③④
Xing 2017 [37]	T2DM	35/35	T: 53.3 ± 6.2 C: 54.1 ± 6.1	T: 18/17 C: 17/18	T: 5.9 ± 2.8 C: 5.8 ± 2.7	Berberine 500 mg tid	Metformin 250 mg tid	12	①③④⑤⑥⑦⑧⑨⑩
Sun 2017 [38]	T2DM	91/91	T: 58.95 ± 10.57 C: 58.34 ± 11.21	T: 53/38 C: 51/40	T: 3.98 ± 1.62 C: 3.64 ± 1.75	C+berberine 30 mg tid	Metformin 0.5 g tid	8	①②③④⑥⑦⑧⑨⑩
Chen et al. 2017 [39]	Prediabetes	50/50	T: 36.5 ± 14.4 C: 35.2 ± 15.1	T: 31/19 C: 30/20	—	Berberine 0.5 g tid	Metformin 0.5 g tid	24	①②⑤
Li et al. 2017 [40]	T2DM	30/30	T: 50.54 ± 3.78 C: 51.24 ± 3.91	T: 18/12 C: 17/13	T: 6.04 ± 2.43 C: 6.61 ± 2.75	C+berberine 0.3 g tid	Sitagliptin 100 mg tid	12	①②③
Li 2016 [41]	T2DM	90/90	T: 56.2 ± 2.9 C: 57.8 ± 2.7	T: 55/35 C: 55/35	T: 3.7 ± 1.9 C: 3.8 ± 1.7	C+berberine 0.3 g tid	Metformin 1.7 g/d	12	①②③④
Lang and Zhu 2016[42]	T2DM	50/50	46.3 ± 4.7	63/37	NA	Berberine 500 mg tid	Placebo	12	①②③
Du 2016 [43]	T2DM	37/36	T: 67.8 ± 4.6 C: 56.5 ± 7.1	T: 21/16 C: 18/18	T: 10.9 ± 6.3 C: 11.6 ± 6.9	C+berberine 4 pills tid	Metformin 0.5 g tid	4	①②③
Li 2016 [44]	T2DM	60/60	T: 65.85 ± 4.78 C: 67.92 ± 4.73	T: 26/24 C: 28/32	T: 5.32 ± 1.08 C: 5.28 ± 1.31	C+berberine 0.5 g tid	Glipizide 5 mg tid	12	③④⑥⑦⑧⑨
Cui 2016 [45]	T2DM	40/40	T: 50.85 ± 7.26 C: 51.59 ± 8.97	T: 28/12 C: 30/10	T: 8.65 ± 3.33 C: 9.07 ± 3.47	C+berberine 0.5 g tid	Metformin 0.5 g tid	16	①②③⑥⑦
Ren et al. 2016 [46]	T2DM	31/30	T: 47.74 ± 7.39 C: 46.87 ± 7.74	T: 21/10 C: 19/11	Newly diagnosed	C+berberine 0.5 g tid	Life interverntion	12	①②③⑥⑦⑧⑨
Wang 2015 [25]	T2DM	39/36	T: 51.6 ± 11.7 C: 53.4 ± 11.3	T: 22/17 C: 21/15	T: 1.7 ± 0.78 C: 1.9 ± 0.71	C+Huanglian 3 g tid	Metformin 0.5 g tid	8	①②③④⑥⑦⑧⑨
Yu 2015 [47]	T2DM	49/48	T: 51.7 ± 4.6 C: 50.9 ± 3.8	T: 23/26 C: 25/23	T: 4.1 ± 0.5 C: 4.3 ± 0.7	C+berberine 0.5 g tid	Metformin 0.5 g qd	12	①③⑥⑦
Zhu et al. 2015 [48]	T2DM	59/59	T: 66.4 ± 7.6 C: 65.6 ± 7.2	T: 35/24 C: 33/26	T: 4.5 ± 1.8 y C: 4.3 ± 2.04	C+berberine 100 mg tid	Gliclazide modified release tablets 30 mg qd	12	①②③⑥⑦⑧⑨
Shu 2014 [49]	T2DM	32/32	T: 62.80 ± 12.20 C: 61.21 ± 13.52	T: 19/13 C: 20/12	T: 6.53 ± 2.61 C: 6.79 ± 2.93	C+berberine 300 mg tid	Metformin 0.5 g tid	24	①④
Zhou 2014 [50]	T2DM	33/33	65.3 ± 2.5	46/20	7.4 ± 1.4	C+berberine 300 mg tid	Metformin 0.5 g bid	12	①②③⑤⑥⑦⑧⑨
Cao 2012 [51]	T2DM	38/40	T: 51.26 ± 14.71 C: 52.53 ± 6.37	T: 22/16 C: 24/16	Newly diagnosed	C+berberine 0.5 g tid	Metformin 0.5 g tid	16	①②③④⑤⑥⑦⑧⑨⑩
Zhou and Huang 2012 [52]	T2DM	45/45	46.67 ± 8.52	48/44	4.81 ± 0.29	C+berberine 0.5 g tid	Metformin 0.5 g tid	12	①②④⑤⑥⑦⑧⑨
Zhang and Yuan 2012 [53]	T2DM	38/38	No difference	T: 21/17 C: 20/18	No difference	C+berberine 0.5 g-0.8 tid	Metformin 0.5 g tid	12	①②③
Xue et al. 2012 [54]	T2DM	44/45	No difference	No difference	No difference	C+berberine 0.5 g tid	Metformin 1.5 g/d SU	12	①②③④
Lou et al. 2011 [55]	T2DM	30/30	36-74	32/28	Newly diagnosed	C+berberine 0.3 g tid	Metformin 1.5 g/d	24	①②③④⑥⑦⑧⑨
Qiu et al. 2011 [56]	T2DM	49/51	T: 42.2 ± 11.4 C: 54 ± 12.5	T: 26/23 C: 28/23	NA	C+berberine 0.3 g tid	Antidiabetic drugs	8	①②③④⑩
Meng et al. [57] 2011	T2DM	30/30	T: 51 ± 13.3 C: 53 ± 13.9	T: 17/13 C: 16/14	Newly diagnosed	C+berberine 0.3 g tid	Insulin injection	12	①②③
Ye 2010 [58]	T2DM	40/40	T: 43∙5 ± 9∙8 C: 42∙5 ± 8∙6	T: 24/16 C: 22/18	T: 0.84 ± 0.36 C: 0.8 ± 0.35	C+berberine 0.5 g tid	Glimepiride 0.1 mg bid Metformin 0.5 g tid	12	①②③⑤⑥⑦⑧⑨
Sheng and Xie 2010 [59]	T2DM	30/30	T: 52 ± 11 C: 51 ± 8	T: 18/12 C: 11/19	T: 5 ± 3 C: 6 ± 3	C+berberine 0.5 g tid	Glipizide 5 mg bid Metformin 0.5 g tid	12	①④⑤⑩
Xu et al. 2008 [60]	T2DM	32/32	43.4 ± 2.1	34/30	Newly diagnosed	C+berberine 0.3 g tid	Pioglitazone 30 mg qd	12	①②⑦
Zhang et al. 2008 [61]	T2DM	59/57	T: 51 ± 9 C: 51 ± 10	T: 30/28 C: 31/21	Newly diagnosed	Berberine 0.5 g bid	Placebo	12	①②③④⑤⑥⑦⑧⑨⑩
Li et al. 2008 [62]	T2DM	33/32	T: 47.5 C: 49.2	T: 17/16 C: 16/16	T: 9.01 ± 1.99 C: 8.11 ± 2.24	Berberine 500 mg tid	Metformin 250 mg tid	12	①②③④⑤⑥⑦⑧⑨⑩
Liu and Hu 2008 [63]	T2DM	30/30	T: 52 ± 9.81 C: 53.07 ± 8.51	T: 10/20 C: 14/16	T: 9.01 ± 1.99 C: 8.11 ± 2.24	C+berberine 0.3-0.5 g tid	Metformin 500 mg tid	8	①②③④
Ju et al. 2007 [23]	Prediabetes	46/44	T: 46.7 ± 4.2 C: 45.6 ± 4.4	T: 28/18 C: 26/18	—	Berberine 0.6 g/d	NA	48	①②⑤⑥⑦⑧⑨⑩
Zhang 2005 [64]	T2DM	48/46	43 ± 11	50/44	6-10	SU+berberine 0.3 g tid	Metformin 1.5 g/d + SU	12	①②③④
Cao 2004 [65]	T2DM	30/30	T: 55.3 ± 11.5 C: 55.4 ± 10.7	T: 13/17 C: 12/18	T: 0.8 ± 0.28 C: 0.825 ± 0.275	Berberine 0.5 g tid Life intervention	Life intervention	12	①②③④⑤⑥⑦⑧⑨⑩

Note: ① FBG: fasting blood glucose; ② 2hPBG: 2-hour postprandial blood glucose; ③ HbA1c: glycated hemoglobin; ④ FINS: fasting insulin; ⑤ BMI: body mass index: ⑥ TC: total cholesterol; ⑦ TG: triglyceride; ⑧ HDL-C: high-density lipoprotein cholesterol; ⑨ LDL-L: low-density lipoprotein cholesterol; ⑩HOMA-IR.

## Data Availability

The original contributions presented in the study are included in the article material; further inquiries can be directed to the corresponding authors.

## Abbreviations

DM: Diabetes mellitus
RCT: Randomized controlled trials
HbA1c: Glycosylated hemoglobin
FPG: Fasting plasm glucose
2hPG: 2-hour postprandial blood glucose
FINS: Fasting blood insulin
HOMA-IR: Homeostasis model assessment-insulin resistance
BMI: Body mass index
TG: Triglyceride
TC: Total cholesterol
LDL: Low-density lipoprotein
HDL: High-density lipoprotein
CRP: C-reactive protein
IL-6: Interleukin-6
TNF-*α*: Tumor necrosis factor-*α*
Scr: Serum creatinine
BUN: Blood urea nitrogen.

## Supplementary Materials

Supplementary 1 Supplementary Files 1 shows the full electronic search strategy for PubMed according to the search history.

Supplementary 2 Supplementary Files 2 shows results of subgroup analyses for main outcomes including HbA1c, FPG, and 2hPG.

Supplementary 3 Supplementary Files 3 shows GRADE criteria to determine the certainty in the estimate of effect for primary outcomes including HbA1c, FPG, and 2hPG.
