# Supplementary material for: The Effect of Berberine on Metabolic Profiles in Type 2 Diabetic Patients: A Systematic Review and Meta-Analysis of Randomized Controlled Trials
Source: Oxid Med Cell Longev. 2021 Dec 15;2021:2074610. doi: 10.1155/2021/2074610 (PMC8696197; doi:10.1155/2021/2074610)
Supplement: Supplementary 1 — Supplementary Files 1 shows the full electronic search strategy for PubMed according to the search history. [file 2074610.f1.pdf]

Queries in pubmed

Search	Query
#45	Search ((((((((((((((((((Medicine, Chinese Traditional[MeSH Terms]) OR Drugs, Chinese Herbal[MeSH Terms]) OR Medicine, Oriental Traditional[MeSH Terms]) OR Plants, Medicinal[MeSH Terms]) OR ((Medicinal Plant[Text Word] OR Plant, Medicinal[Text Word] OR Medicinal Plants[Text Word] OR Pharmaceutical Plants[Text Word] OR Pharmaceutical Plant[Text Word] OR Plant, Pharmaceutical[Text Word] OR Plants, Pharmaceutical[Text Word] OR Healing Plants,[Text Word] OR Healing Plant[Text Word] OR Plant, Healing[Text Word] OR Plants, Healing[Text Word] OR Medicinal Herbs[Text Word] OR Herb, Medicinal[Text Word] OR Medicinal Herb[Text Word] OR Herbs, Medicinal[Text Word]))) OR ((Oriental Medicine, Traditional[Text Word] OR Medicine, Traditional Oriental[Text Word] OR Traditional Oriental Medicine[Text Word] OR Traditional Oriental Medicines[Text Word] OR Traditional Medicine, Oriental[Text Word] OR Traditional East Asian Medicine[Text Word] OR Medicine, Traditional, East Asia[Text Word] OR Traditional Medicine, East Asia[Text Word] OR Traditional Far Eastern Medicine[Text Word] OR East Asian Traditional Medicine[Text Word] OR Oriental Traditional Medicine[Text Word] OR Medicine, Oriental Traditional[Text Word] OR East Asian Medicine[Text Word] OR East Asian Medicines[Text Word] OR Medicine, East Asian[Text Word] OR Oriental Medicine[Text Word] OR Medicine, Far East[Text Word] OR East Medicine, Far[Text Word] OR East Medicines, Far[Text Word] OR Far East Medicine[Text Word] OR Far East Medicines[Text Word] OR Medicines, Far East[Text Word] OR Medicine, East Asia[Text Word] OR Asia Medicines, East[Text Word] OR East Asia Medicine[Text Word] OR East Asia Medicines[Text Word] OR Medicines, East Asia[Text Word] OR Medicine, Oriental[Text Word]))) OR ((Chinese Drugs, Plant[Text Word] OR Chinese Herbal Drugs[Text Word] OR Herbal Drugs, Chinese[Text Word] OR Plant Extracts, Chinese[Text Word] OR Chinese Plant Extracts[Text Word] OR Extracts, Chinese Plant[Text Word]))) OR ((Traditional Chinese Medicine[Text
#44	Search (((((((Controlled Clinical Trial[Publication Type]) OR randomized controlled trial[Publication Type]) OR randomized controlled trials[MeSH Terms]) OR random allocation[MeSH Terms]) OR double-blind method[MeSH Terms]) OR single-blind method[MeSH Terms]) OR clinical study[Title/Abstract]) OR (((clinical trial[Title/Abstract] OR Controlled Clinical
#43	Search ((clinical trial[Title/Abstract] OR Controlled Clinical Trial[Title/Abstract] OR randomized controlled trial[Title/Abstract] OR
#41	Search clinical study[Title/Abstract]
#38	Search single-blind method[MeSH Terms]
#37	Search double-blind method[MeSH Terms]
#36	Search random allocation[MeSH Terms]
#35	Search randomized controlled trials[MeSH Terms]
#34	Search randomized controlled trial[Publication Type]
#33	Search Controlled Clinical Trial[Publication Type]

#27	Search ((((((((((((((((Medicine, Chinese Traditional[MeSH Terms]) OR Drugs, Chinese Herbal[MeSH Terms]) OR Medicine, Oriental Traditional[MeSH Terms]) OR Plants, Medicinal[MeSH Terms]) OR ((Medicinal Plant[Text Word] OR Plant, Medicinal[Text Word] OR Medicinal Plants[Text Word] OR Pharmaceutical Plants[Text Word] OR Pharmaceutical Plant[Text Word] OR Plant, Pharmaceutical[Text Word] OR Plants, Pharmaceutical[Text Word] OR Healing Plants,[Text Word] OR Healing Plant[Text Word] OR Plant, Healing[Text Word] OR Plants, Healing[Text Word] OR Medicinal Herbs[Text Word] OR Herb, Medicinal[Text Word] OR Medicinal Herb[Text Word] OR Herbs, Medicinal[Text Word]))) OR ((Oriental Medicine, Traditional[Text Word] OR Medicine, Traditional Oriental[Text Word] OR Traditional Oriental Medicine[Text Word] OR Traditional Oriental Medicines[Text Word] OR Traditional Medicine, Oriental[Text Word] OR Traditional East Asian Medicine[Text Word] OR Medicine, Traditional, East Asia[Text Word] OR Traditional Medicine, East Asia[Text Word] OR Traditional Far Eastern Medicine[Text Word] OR East Asian Traditional Medicine[Text Word] OR Oriental Traditional Medicine[Text Word] OR Medicine, Oriental Traditional[Text Word] OR East Asian Medicine[Text Word] OR East Asian Medicines[Text Word] OR Medicine, East Asian[Text Word] OR Oriental Medicine[Text Word] OR Medicine, Far East[Text Word] OR East Medicine, Far[Text Word] OR East Medicines, Far[Text Word] OR Far East Medicine[Text Word] OR Far East Medicines[Text Word] OR Medicines, Far East[Text Word] OR Medicine, East Asia[Text Word] OR Asia Medicines, East[Text Word] OR East Asia Medicine[Text Word] OR East Asia Medicines[Text Word] OR Medicines, East Asia[Text Word] OR Medicine, Oriental[Text Word]))) OR ((Chinese Drugs, Plant[Text Word] OR Chinese Herbal Drugs[Text Word] OR Herbal Drugs, Chinese[Text Word] OR Plant Extracts, Chinese[Text Word] OR Chinese Plant Extracts[Text Word] OR Extracts, Chinese Plant[Text Word]))) OR ((Traditional Chinese Medicine[Text

#26	Search ((((((diabetes mellitus, non-insulin-dependent[MeSH Terms]) OR insulin resistance[MeSH Terms]) OR Metabolic Syndrome[MeSH Terms]) OR ((Diabetes Mellitus, Noninsulin-Dependent[Text Word] OR Diabetes Mellitus, Ketosis-Resistant[Text Word] OR Diabetes Mellitus, Ketosis Resistant[Text Word] OR Ketosis-Resistant Diabetes Mellitus[Text Word] OR Diabetes Mellitus, Non Insulin Dependent[Text Word] OR Diabetes Mellitus, Non-Insulin-Dependent[Text Word] OR Non-Insulin-Dependent Diabetes Mellitus[Text Word] OR Diabetes Mellitus, Stable[Text Word] OR Stable Diabetes Mellitus[Text Word] OR Diabetes Mellitus, Type II[Text Word] OR NIDDM[Text Word] OR Diabetes Mellitus, Noninsulin Dependent[Text Word] OR Diabetes Mellitus, Maturity-Onset[Text Word] OR Diabetes Mellitus, Maturity Onset[Text Word] OR Maturity-Onset Diabetes Mellitus[Text Word] OR Maturity Onset Diabetes Mellitus[Text Word] OR MODY[Text Word] OR Diabetes Mellitus, Slow-Onset[Text Word] OR Diabetes Mellitus, Slow Onset[Text Word] OR Slow-Onset Diabetes Mellitus[Text Word] OR Type 2 Diabetes Mellitus[Text Word] OR Noninsulin-Dependent Diabetes Mellitus[Text Word] OR Noninsulin Dependent Diabetes Mellitus[Text Word] OR Maturity-Onset Diabetes[Text Word] OR Diabetes, Maturity-Onset[Text Word] OR Maturity Onset Diabetes[Text Word] OR Type 2 Diabetes[Text Word] OR Diabetes, Type 2[Text Word] OR Diabetes Mellitus, Adult-Onset[Text Word] OR Adult-Onset Diabetes Mellitus[Text Word] OR Diabetes Mellitus, Adult Onset[Text Word]))) OR (Search (insulin resistance[Text Word] OR Resistance, Insulin[Text Word] OR Insulin Sensitivity[Text Word] OR Sensitivity, Insulin[Text Word]))) OR (((insulin resistance[Text Word] OR Resistance, Insulin[Text Word] OR Insulin Sensitivity[Text Word] OR Sensitivity, Insulin[Text Word])))) OR ((Metabolic Syndrome[Text Word] OR Metabolic Syndromes[Text Word] OR Syndrome, Metabolic[Text Word] OR
#25	Search (Metabolic Syndrome[Text Word] OR Metabolic Syndromes[Text Word] OR Syndrome, Metabolic[Text Word] OR Syndromes, Metabolic[Text Word] OR Metabolic Syndrome X[Text Word] OR Insulin Resistance Syndrome X[Text Word] OR Syndrome X, Metabolic[Text Word] OR Syndrome X, Insulin Resistance[Text Word] OR Metabolic X Syndrome[Text Word] OR Syndrome, Metabolic X[Text Word] OR X Syndrome, Metabolic[Text Word] OR Dysmetabolic Syndrome X[Text Word] OR Syndrome X, Dysmetabolic[Text Word] OR Reaven Syndrome X[Text Word]
#24	Search ((insulin resistance[Text Word] OR Resistance, Insulin[Text Word] OR Insulin Sensitivity[Text Word] OR Sensitivity, Insulin[Text Word]))
#23	Search Search (insulin resistance[Text Word] OR Resistance, Insulin[Text Word] OR Insulin Sensitivity[Text Word] OR Sensitivity, Insulin[Text Word])

#22	Search (Diabetes Mellitus, Noninsulin-Dependent[Text Word] OR Diabetes Mellitus, Ketosis-Resistant[Text Word] OR Diabetes Mellitus, Ketosis Resistant[Text Word] OR Ketosis-Resistant Diabetes Mellitus[Text Word] OR Diabetes Mellitus, Non Insulin Dependent[Text Word] OR Diabetes Mellitus, Non-Insulin-Dependent[Text Word] OR Non-Insulin-Dependent Diabetes Mellitus[Text Word] OR Diabetes Mellitus, Stable[Text Word] OR Stable Diabetes Mellitus[Text Word] OR Diabetes Mellitus, Type II[Text Word] OR NIDDM[Text Word] OR Diabetes Mellitus, Noninsulin Dependent[Text Word] OR Diabetes Mellitus, Maturity-Onset[Text Word] OR Diabetes Mellitus, Maturity Onset[Text Word] OR Maturity-Onset Diabetes Mellitus[Text Word] OR Maturity Onset Diabetes Mellitus[Text Word] OR MODY[Text Word] OR Diabetes Mellitus, Slow-Onset[Text Word] OR Diabetes Mellitus, Slow Onset[Text Word] OR Slow-Onset Diabetes Mellitus[Text Word] OR Type 2 Diabetes Mellitus[Text Word] OR Noninsulin-Dependent Diabetes
#21	Search Metabolic Syndrome[MeSH Terms]
#20	Search insulin resistance[MeSH Terms]
#19	Search diabetes mellitus, non-insulin-dependent[MeSH Terms]
#18	Search ((((((((((((((Medicine, Chinese Traditional[MeSH Terms]) OR Drugs, Chinese Herbal[MeSH Terms]) OR Medicine, Oriental Traditional[MeSH Terms]) OR Plants, Medicinal[MeSH Terms]) OR ((Medicinal Plant[Text Word] OR Plant, Medicinal[Text Word] OR Medicinal Plants[Text Word] OR Pharmaceutical Plants[Text Word] OR Pharmaceutical Plant[Text Word] OR Plant, Pharmaceutical[Text Word] OR Plants, Pharmaceutical[Text Word] OR Healing Plants,[Text Word] OR Healing Plant[Text Word] OR Plant, Healing[Text Word] OR Plants, Healing[Text Word] OR Medicinal Herbs[Text Word] OR Herb, Medicinal[Text Word] OR Medicinal Herb[Text Word] OR Herbs, Medicinal[Text Word]))) OR ((Oriental Medicine, Traditional[Text Word] OR Medicine, Traditional Oriental[Text Word] OR Traditional Oriental Medicine[Text Word] OR Traditional Oriental Medicines[Text Word] OR Traditional Medicine, Oriental[Text Word] OR Traditional East Asian Medicine[Text Word] OR Medicine, Traditional, East Asia[Text Word] OR Traditional Medicine, East Asia[Text Word] OR Traditional Far Eastern Medicine[Text Word] OR East Asian Traditional Medicine[Text Word] OR Oriental Traditional Medicine[Text Word] OR Medicine, Oriental Traditional[Text Word] OR East Asian Medicine[Text Word] OR East Asian Medicines[Text Word] OR Medicine, East Asian[Text Word] OR Oriental Medicine[Text Word] OR Medicine, Far East[Text Word] OR East Medicine, Far[Text Word] OR East Medicines, Far[Text Word] OR Far East Medicine[Text Word] OR Far East Medicines[Text Word] OR Medicines, Far East[Text Word] OR Medicine, East Asia[Text Word] OR Asia Medicines, East[Text Word] OR East Asia Medicine[Text Word] OR East Asia Medicines[Text Word] OR Medicines, East Asia[Text Word] OR Medicine, Oriental[Text Word]))) OR ((Chinese Drugs, Plant[Text Word] OR Chinese Herbal Drugs[Text Word] OR Herbal Drugs, Chinese[Text Word] OR Plant Extracts, Chinese[Text Word] OR Chinese Plant Extracts[Text Word] OR Extracts, Chinese Plant[Text Word]))) OR ((Traditional Chinese Medicine[Text
#17	Search (huang*lian*su[Text Word] OR xiao*bo* jian[Text Word])
#16	Search Rhizoma coptidis[Text Word]
#15	Search Coptis chinensis[Text Word]
#14	Search Copti[Text Word] OR Goldthread[Text Word] OR Goldthreads[Text
#13	Search huanglian[MeSH Terms]

#12	Search Coptis[MeSH Terms]
#11	Search berberine[MeSH Terms]
#10	Search (Medicine, Chinese Traditional[Text Word] OR Drugs, Chinese Herbal[Text Word] OR Medicine, Oriental Traditional[Text Word] OR Plants,
#9	Search (Traditional Chinese Medicine[Text Word] OR Chung I Hsueh[Text Word] OR Hsueh, Chung I[Text Word] OR Traditional Medicine, Chinese[Text Word] OR Zhong Yi Xue[Text Word] OR Chinese Traditional Medicine[Text Word] OR Chinese Medicine, Traditional[Text Word] OR Traditional Tongue Diagnosis[Text Word] OR Tongue Diagnoses, Traditional[Text Word] OR Tongue Diagnosis, Traditional[Text Word] OR Traditional Tongue Diagnoses[Text Word] OR Traditional Tongue Assessment[Text Word] OR
#8	Search (Chinese Drugs, Plant[Text Word] OR Chinese Herbal Drugs[Text Word] OR Herbal Drugs, Chinese[Text Word] OR Plant Extracts, Chinese[Text Word] OR Chinese Plant Extracts[Text Word] OR Extracts, Chinese Plant[Text
#7	Search (Oriental Medicine, Traditional[Text Word] OR Medicine, Traditional Oriental[Text Word] OR Traditional Oriental Medicine[Text Word] OR Traditional Oriental Medicines[Text Word] OR Traditional Medicine, Oriental[Text Word] OR Traditional East Asian Medicine[Text Word] OR Medicine, Traditional, East Asia[Text Word] OR Traditional Medicine, East Asia[Text Word] OR Traditional Far Eastern Medicine[Text Word] OR East Asian Traditional Medicine[Text Word] OR Oriental Traditional Medicine[Text Word] OR Medicine, Oriental Traditional[Text Word] OR East Asian Medicine[Text Word] OR East Asian Medicines[Text Word] OR Medicine, East Asian[Text Word] OR Oriental Medicine[Text Word] OR Medicine, Far East[Text Word] OR East Medicine, Far[Text Word] OR East Medicines, Far[Text Word] OR Far East Medicine[Text Word] OR Far East
#6	Search (Medicinal Plant[Text Word] OR Plant, Medicinal[Text Word] OR Medicinal Plants[Text Word] OR Pharmaceutical Plants[Text Word] OR Pharmaceutical Plant[Text Word] OR Plant, Pharmaceutical[Text Word] OR Plants, Pharmaceutical[Text Word] OR Healing Plants,[Text Word] OR Healing Plant[Text Word] OR Plant, Healing[Text Word] OR Plants, Healing[Text
#5	Search Plants, Medicinal[MeSH Terms]
#4	Search Medicine, Oriental Traditional[MeSH Terms]
#3	Search Drugs, Chinese Herbal[MeSH Terms]
#2	Search Medicine, Chinese Traditional[MeSH Terms]
