# Supplementary material for: The Effect of Berberine on Metabolic Profiles in Type 2 Diabetic Patients: A Systematic Review and Meta-Analysis of Randomized Controlled Trials
Source: Oxid Med Cell Longev. 2021 Dec 15;2021:2074610. doi: 10.1155/2021/2074610 (PMC8696197; doi:10.1155/2021/2074610)
Supplement: Supplementary 2 — Supplementary Files 2 shows results of subgroup analyses for main outcomes including HbA1c, FPG, and 2hPG. [file 2074610.f2.docx]

| Subgroups | n | WMD | 95%CI | p value | Heterogeneity Between Studies |
| --- | --- | --- | --- | --- | --- |
| HbAC1 |  |  |  |  |  |
| Daily dosage (g/d) | 38 |  |  |  |  |
| <1 | 16 | -1 | (-1.38,-0.63) | <0.05 | 91.8% |
| 1-2 | 18 | -0.94 | (-1.31,-0.57) | <0.05 | 91.5% |
| >2 | 4 | -0.22 | (-0.69,0.16) | 0.039 | 64.1% |
| Duration(weeks) | 39 |  |  |  |  |
| ≤8 | 7 | -1.62 | (-2.57,-0.66) | <0.05 | 96.9% |
| 8-12 | 23 | -0.62 | (-0.82, 0.41) | <0.05 | 80.9% |
| >12 | 9 | -1.14 | (-1.76, -0.51) | <0.05 | 93.6% |
| Course(years) | 31 |  |  |  |  |
| <5 | 15 | -0.61 | (-0.9,-0.32) | <0.05 | 86.9% |
| 5-10 | 15 | -1.21 | (-1.75,0.66) | <0.05 | 94.7% |
| >10 | 1 | -0.53 | (-1.1,-0.62) | <0.05 | -- |
|  |  |  |  |  |  |
| FBG |  |  |  |  |  |
| Daily dosage (g/d) | 40 |  |  |  |  |
| <1 | 16 | -1.21 | (-1.68,-0.73) | <0.05 | 94% |
| 1-2 | 20 | -0.97 | (-1.35,-0.58) | <0.05 | 92.7% |
| >2 | 4 | -0.19 | (-0.5,0.11) | 0.142 | 44.9% |
| Duration(weeks) | 42 |  |  |  |  |
| ≤8 | 6 | -1.17 | (-1.92,-0.43) | <0.05 | 93.3% |
| 8-12 | 28 | -0.99 | (-1.35, 0.63) | <0.05 | 93.9% |
| >12 | 8 | -0.63 | (-0.94, -0.32) | <0.05 | 75.4% |
| Course(years) | 34 |  |  |  |  |
| <5 | 18 | -1.18 | (-1.77,0.60) | <0.05 | 93.9% |
| 5-10 | 16 | -0.75 | (-1.15,0.34) | <0.05 | 91.4% |
| >10 | 1 | -0.92 | (-1.17,-0.67) | <0.05 | -- |
| 2hPG |  |  |  |  |  |
| Daily dosage (g/d) | 33 |  |  |  |  |
| <1 | 16 | -0.99 | (-1.33,-0.65) | <0.05 | 90.2% |
| 1-2 | 13 | -0.99 | (-1.44,-0.54) | <0.05 | 91.8% |
| >2 | 4 | -0.25 | (-0.68,0.18) | 0.012 | 72.7% |
| Duration(weeks) | 35 |  |  |  |  |
| ≤8 | 6 | -0.46 | (-0.83,-0.09) | <0.05 | 79.9% |
| 8-12 | 22 | -0.93 | (-1.29, -0.58) | <0.05 | 92.6% |
| >12 | 8 | -0.84 | (-1.25, -0.43) | <0.05 | 85.2% |
| Course(years) | 28 |  |  |  |  |
| <5 | 16 | -0.66 | (-0.93,-0.4) | <0.05 | 83.9% |
| 5-10 | 11 | -0.77 | (-1.26,0.29) | <0.05 | 91.8% |
| >10 | 1 | -0.8 | (-1.04,-0.57) | <0.05 | -- |
