# Supplementary figures and images for: The Effect of Berberine on Metabolic Profiles in Type 2 Diabetic Patients: A Systematic Review and Meta-Analysis of Randomized Controlled Trials
Source: Oxid Med Cell Longev. 2021 Dec 15;2021:2074610. doi: 10.1155/2021/2074610 (PMC8696197; doi:10.1155/2021/2074610)

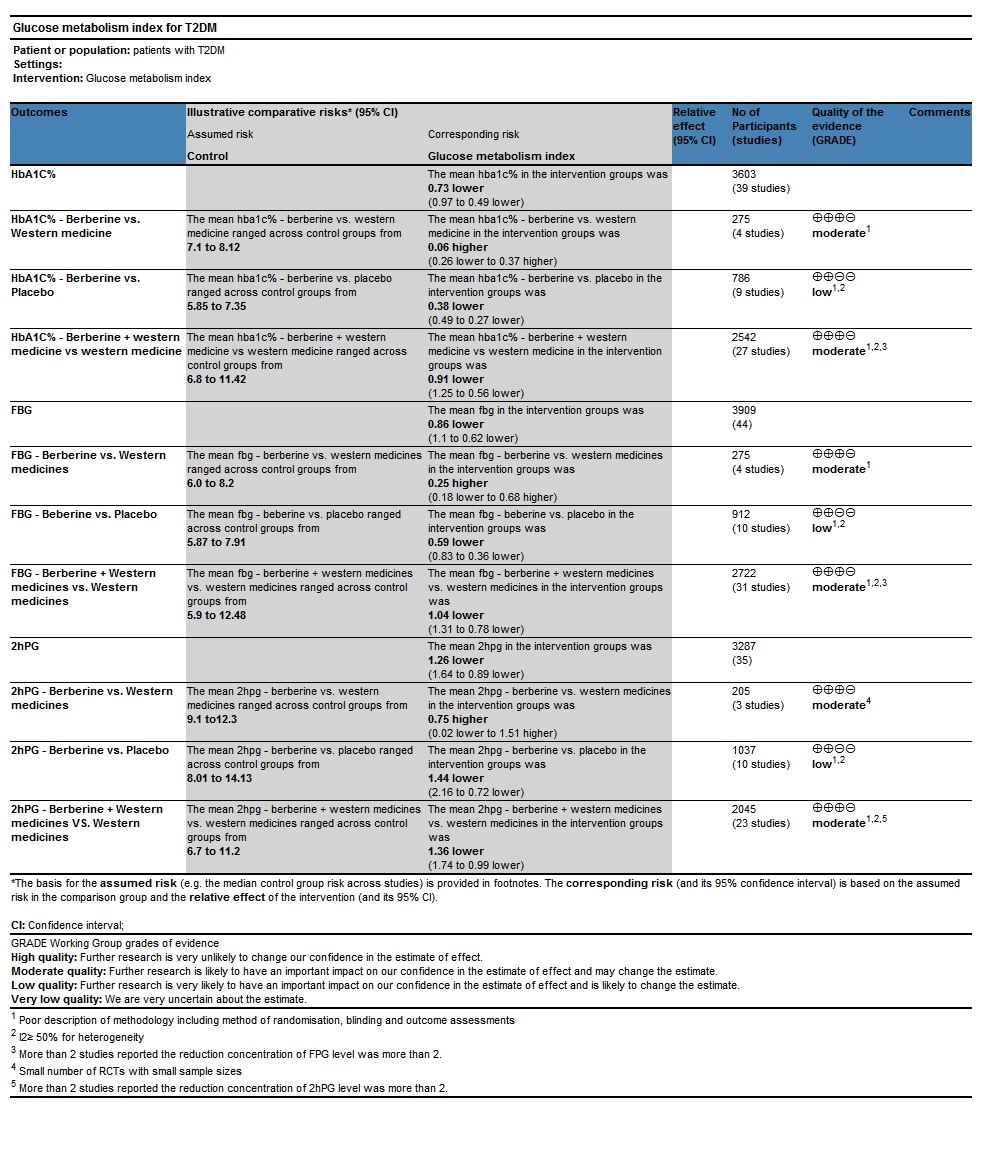

Supplement: Supplementary 3 — Supplementary Files 3 shows GRADE criteria to determine the certainty in the estimate of effect for primary outcomes including HbA1c, FPG, and 2hPG. [file 2074610.f3.docx]
